# Stable *atrogin-1* (*Fbxo32*) and *MuRF1* (*Trim63*) gene expression is involved in the protective mechanism in soleus muscle of hibernating Daurian ground squirrels (*Spermophilus dauricus*)

**DOI:** 10.1242/bio.015776

**Published:** 2016-01-06

**Authors:** Kai Dang, Ya-Zhao Li, Ling-Chen Gong, Wei Xue, Hui-Ping Wang, Nandu Goswami, Yun-Fang Gao

**Affiliations:** 1 Key Laboratory of Resource Biology and Biotechnology in Western China (Northwest University), Ministry of Education, Xi'an 710069, China; 2 Gravitational Physiology and Medicine Research Unit, Institute of Physiology, Center of Physiological Medicine, Medical University Graz, Graz 8010, Austria

**Keywords:** Disuse muscle atrophy, Hibernation, Hindlimb-unloading, Seasons

## Abstract

Understanding the mechanisms that protect against or limit muscle atrophy in hibernators during prolonged inactivity has important implications for its treatment. We examined whether external factors influence the pathways regulating protein synthesis and degradation, leading to muscle atrophy prevention in Daurian ground squirrels (*Spermophilus dauricus*). We investigated the effects of 14-day hindlimb-unloading (HU) in different seasons and two-month hibernation on the soleus (SOL) muscle wet mass, muscle-to-body mass ratio, fiber cross sectional area (CSA), fiber distribution and muscle ultrastructure. We also measured changes in the protein expression and activation states of Akt, mTOR and FoxO1 and the mRNA expression of *atrogin-1* and *MuRF1*. Compared with the control groups, autumn and winter HU significantly lowered SOL muscle wet mass and muscle-to-body mass ratio, decreased type I and II fiber CSA and induced ultrastructural anomalies. However, these measured indices were unchanged between Pre-hibernation and Hibernation groups. Furthermore, phosphorylation levels of Akt and mTOR significantly decreased, while the phosphorylation level of FoxO1 and mRNA expression of *atrogin-1* and *MuRF1* increased after HU. During hibernation, the phosphorylation levels of Akt and mTOR significantly decreased, but the phosphorylation level of FoxO1 and mRNA expression of *atrogin-1* and *MuRF1* remained unchanged. Overall, our findings suggest that disuse and seasonality may not be sufficient to initiate the innate protective mechanism that prevents SOL atrophy during prolonged periods of hibernation inactivity. The stable expression of atrogin-1 and MuRF1 may facilitate to prevent SOL atrophy via controlling ubiquitination of muscle proteins during hibernation.

## Abstract (precis)

**Summary:** mRNA expression of *atrogin-1* and *MuRF1* remains unchanged during hibernation of Daurian ground squirrels, suggesting that stable expression of these genes may facilitate the prevention of SOL atrophy via controlling ubiquitylation of muscle proteins during hibernation.

## INTRODUCTION

As the largest organ in the human body, skeletal muscle makes up about 40 percent of total human body weight. Maintenance of muscle mass and function is essential for health and survival. Adaptive changes quickly occur in mammalian skeletal muscle under reduced activity. Hindlimb suspension or immobilization leads to rapid atrophy in rat skeletal muscles, characterized by decreased muscle mass, shrinking fiber size (fiber cross sectional area, CSA) and transformation from slow-twitch to fast-twitch fibers (Desplanches et al., 1987; Kourtidou-Papadeli et al., 2004; Guillot et al., 2008; Du et al., 2011). Disuse muscle atrophy has also been observed in human studies during limb immobilization, bed rest and space travel (Adams et al., 2003; di Prampero and Narici, 2003; Dilani Mendis et al., 2009).

Disuse muscle atrophy not only generates changes in muscle morphologies, but also increases functional deterioration (e.g. reduction in force production) (Degens and Alway, 2006), resulting in a negative effect on quality of life. Despite its commonality worldwide and decades of research, no suitable treatments have been found to effectively prevent or cure the loss of muscle mass and contractile tension following physical inactivity. Lack of knowledge regarding the molecular mechanisms involved in muscle loss and function degradation during physical inactivity may explain why appropriate treatment strategies remain unrealized.

Hibernation is an important survival strategy by which hibernators overcome the challenges of low temperature and limited food availability (Carey et al., 2003). Despite facing long-term inactivity, hibernators are capable of preventing disuse muscle atrophy, and loss of skeletal muscle is minimal or absent during hibernation (Lohuis et al., 2007; Lee et al., 2008; Gao et al., 2012; Bodine, 2013b). Due to their innate ability to prevent muscle atrophy under conditions of prolonged disuse, hibernating animals are commonly used as models to study the protective mechanisms involved in skeletal muscles. Understanding such mechanisms has important implications for the treatment of disuse muscle atrophy. However, the molecular mechanisms preventing atrophy in hibernating mammals have not yet been elucidated.

The preservation of muscle mass depends on a homeostatic balance between protein synthesis and degradation. Although muscle atrophy has multifactorial etiology, current understanding points towards a negative protein balance as the major contributor (Glass, 2005; Bodine, 2013a). Specifically, the Akt-mTOR pathway is currently considered the principal signaling protein cascade regulating protein synthesis (Bodine et al., 2001a; Glass, 2005; Léger et al., 2006). Indeed, researchers have shown that insulin-like growth factor 1 (IGF1) regulates muscle growth via activation of this pathway (Rommel et al., 2001; Glass, 2005). By phosphorylating serine/threonine kinase Akt (protein kinase B), activated phosphoinositide 3-kinase (PI3K) initiates the Akt-mTOR pathway. Akt is a key regulator of several signaling pathways associated with skeletal muscle homeostasis, and its major direct target downstream is the mammalian target of rapamycin (mTOR) kinase. This kinase consists of two independent multiprotein complexes, mTORC1 and mTORC2, which have different properties and functions (Drummond et al., 2009; Risson et al., 2009). The mTORC1 complex promotes protein synthesis via the phosphorylation of eukaryotic translation initiation factor 4E (eIF4E) binding protein 1 (4E-BP1) and p70 ribosomal S6 kinase (p70^S6K^) (Ma and Blenis, 2009). Inhibition of the Akt/mTOR pathway leads to muscle atrophy (Hoffman and Nader, 2004).

**List of symbols and abbreviations**

A-CON: Autumn-control
A-HU: Autumn-HU
CSA: Cross sectional area
HIB: Hibernation
HU: Hindlimb-unloading
PRE: Pre-hibernation
SOL: Soleus
W-CON: Winter-control
W-HU: Winter-HU
#: Compared to Pre-hibernation, *P*<0.05
*: Compared to control group*, P*<0.05

Apart from protein synthesis suppression, disuse muscle atrophy is also caused by activation of the protein degradation pathways. The ubiquitin proteasome pathway (UPP) plays a central role in protein degradation following disuse (Solomon and Goldberg, 1996; Taillandier et al., 1996). The UPP involves three ubiquitin ligase enzymes (i.e. E1–E3), which attach ubiquitin cofactors to proteins, leading to their degradation by the proteasome system (Attaix et al., 2005). As identified muscle-specific E3 ligases, atrogin and muscle RING finger 1 (MuRF1) are regulated by members of the FoxO family, and their mRNA expression levels are reportedly increased in response to disuse muscle atrophy (Bodine et al., 2001b; Sandri et al., 2004; Sacheck et al., 2007).

The purpose of this study was to investigate the potential protective mechanism against disuse muscle atrophy in hibernators by determining which putative external factors are involved in triggering this mechanism and which signaling pathways are implicated. We conducted 14-day hindlimb-unloading (HU) of Daurian ground squirrels (*Spermophilus dauricu*s) in order to compare experimental HU and unrestrained hibernation. It has been well documented that muscle atrophy is muscle-type specific in hibernators, while the soleus (SOL), which is composed predominantly of type I fibers, is protected from atrophy during hibernation (Wickler et al., 1991; Nowell et al., 2011; Hindle et al., 2015). Therefore, the SOL is an ideal focus for comparison. We investigated the effects of 14-day HU in different seasons and two-month hibernation on SOL muscle wet mass, muscle-to-body mass ratio, fiber CSA, fiber distribution and muscle ultrastructure. We also measured changes in the protein expression and activation states of Akt, mTOR and FoxO1 and the mRNA expression of *atrogin-1* and *MuRF1* after HU and hibernation.

## RESULTS

### Effects of hindlimb unloading in different seasons and hibernation on SOL muscle wet mass, muscle-to-body mass ratio, fiber CSA and fiber distribution of Daurian ground squirrels

Muscle atrophy is characterized by loss of muscle mass and decrease in fiber CSA and is accompanied by a shift from slow-twitch (type I) to fast-twitch (type II) fibers in non-hibernating mammals (Adams et al., 2003; Guillot et al., 2008; Bodine, 2013a). To evaluate the effects of HU in different seasons and hibernation on Daurian ground squirrel SOL, we investigated SOL muscle wet mass, muscle-to-body mass ratio, fiber CSA and fiber distribution.

**Fig. 1.**
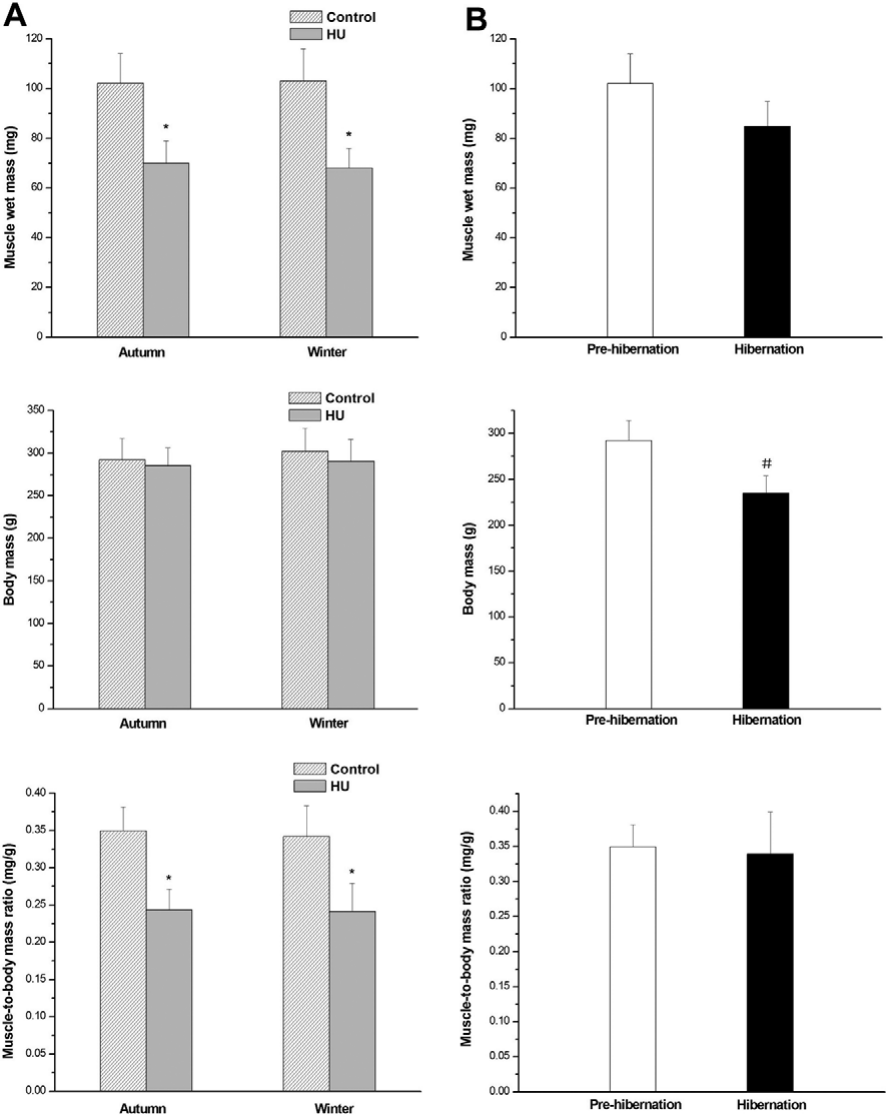
**Effects of HU in different seasons and hibernation on SOL muscle wet mass and muscle-to-body-mass ratio in Daurian ground squirrels.** (A) Changes in SOL muscle-to-body-mass ratio after HU in different seasons (*N*=8 each; one-way ANOVA), showing that HU significantly lowered muscle wet mass and muscle-to-body mass ratio. (B) Changes in SOL muscle-to-body-mass ratio during hibernation (*N*=8 each; two-tailed independent sample t-test), showing that hibernation significantly lowered body mass, but did not affect muscle wet mass and muscle-to-body mass ratio. *P<0.05, Compared with control group; #*P*<0.05, Compared with Pre-hibernation. Data are expressed as means±s.d.

Compared with the controls, SOL muscle wet mass and muscle-to-body-mass ratio were lower in the Autumn-HU (−32% and −30%, respectively) and Winter-HU groups (−32% and −31%, respectively) (all *P*<0.05) (Fig. 1A,C,E). However, the muscle wet mass and muscle-to-body mass ratio did not statistically differ between the Autumn-control and Winter-control groups or between the Pre-hibernation and Hibernation groups (Fig. 1B,D,F).

**Fig. 2.**
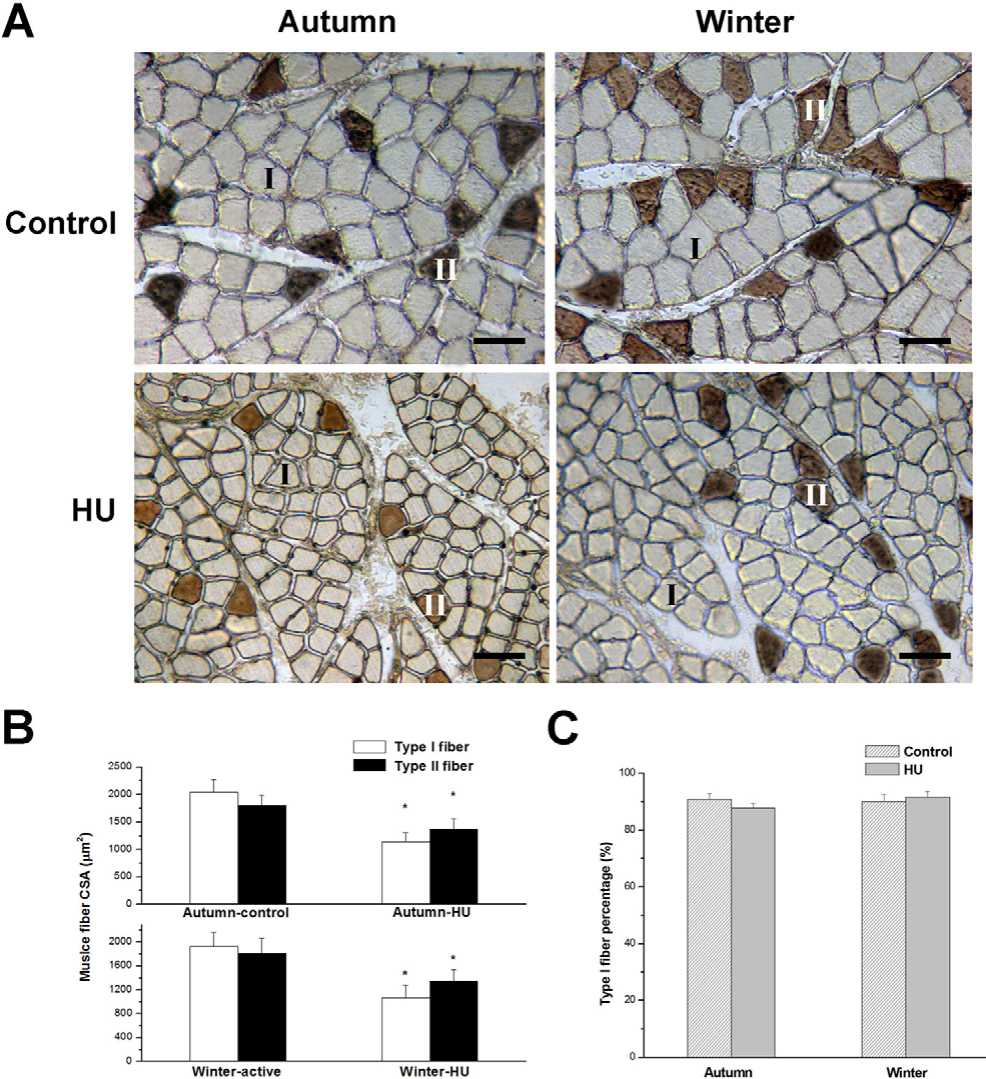
**Effects of HU in different seasons on SOL fiber cross-sectional area (CSA) and fiber distribution in Daurian ground squirrels.** (A) Serial sections immunostained with antibody MY32 against Myosin heavy chain II (MHC II). (B,C) Changes in SOL fiber CSA (B) and fiber distribution (C) after HU in different seasons, showing that HU significantly decreased fiber CSA, but did not affect fiber distribution. *P<0.05, compared with control group. Scale bars=60 µm. *N*=8 each, one-way ANOVA. Data are expressed as means±s.d.

**Fig. 3.**
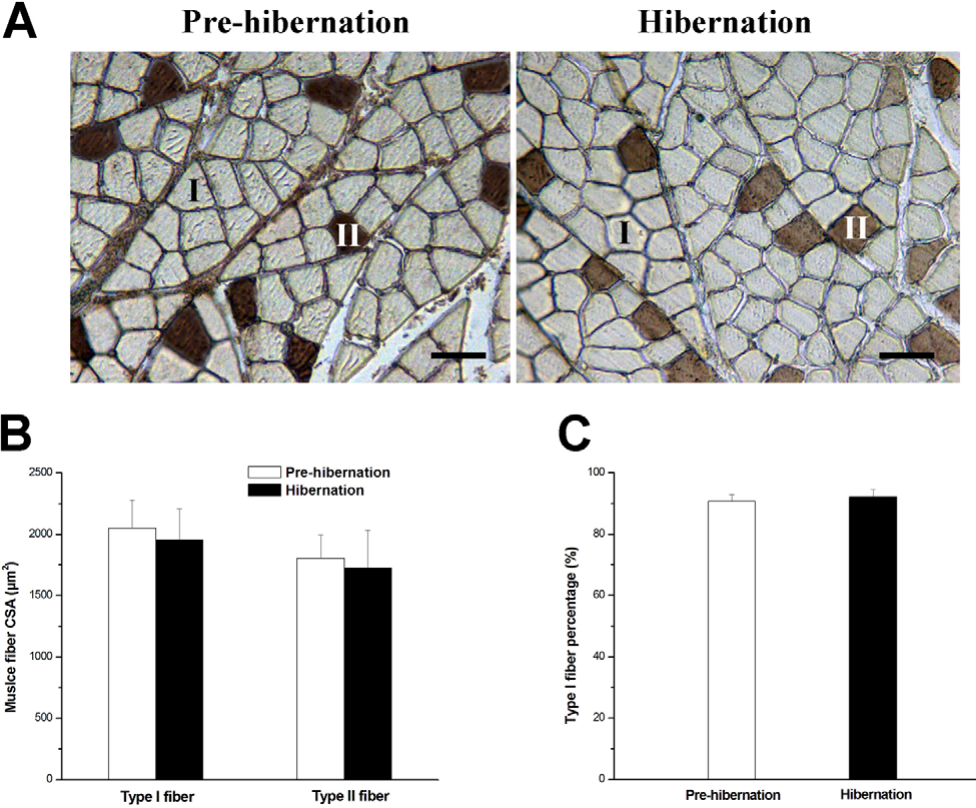
**Effect of hibernation on SOL fiber cross-sectional area (CSA) and fiber distribution in Daurian ground squirrels.** (A) Serial sections immunostained with antibody MY32 against Myosin heavy chain II (MHC II). (B,C) Changes in SOL fiber CSA (B) and fiber distribution (C) during hibernation, showing no effect of hibernation on fiber CSA and fiber distribution. Scale bars=60 μm. *N*=8 each, two-tailed independent sample t-test. Data are expressed as means±s.d.

HU in different seasons statistically decreased type I and II fiber CSA. Compared with the control groups, type I fiber CSA decreased by 44% (*P*<0.05) in the Autumn-HU group and by 46% (*P*<0.05) in the Winter-HU. The corresponding decreases in type II fiber were 24% (*P*<0.05) and 29% (*P*<0.05), respectively. However, no significant differences in muscle fiber CSA between the Autumn-control and Winter-control groups were observed (Fig. 2A,B). Type I and type II fiber CSA in the Hibernation group were 1953±255 μm^2^ and 1723±310 μm^2^, respectively. When compared with the Pre-hibernation group, type I and II fibers did not vary significantly (Fig. 3A,B).

In the HU group, type I fiber percentages were 87.6±1.8 (Autumn) and 91.5±2.0% (Winter), respectively. Compared with the controls, there was no intergroup statistical difference. In addition, there were no statistical differences between the Autumn-control and Winter-control groups (Fig. 2A,C). Type I fiber percentages in the Pre-hibernation and Hibernation groups were 90.7±2.2% and 92.1±2.4%, respectively. There was no statistical difference in fiber distribution between the groups (Fig. 3A,C).

SOL muscle wet mass, muscle-to-body mass ratio, and type I and II CSA decreased in both HU groups and remained unchanged in the Hibernation group, indicating that the SOL was atrophied during 14-day HU in autumn and winter, but protected during the two -month hibernation.

### Effects of hindlimb unloading in different seasons and hibernation on SOL ultrastructure of Daurian ground squirrels

**Fig. 4.**
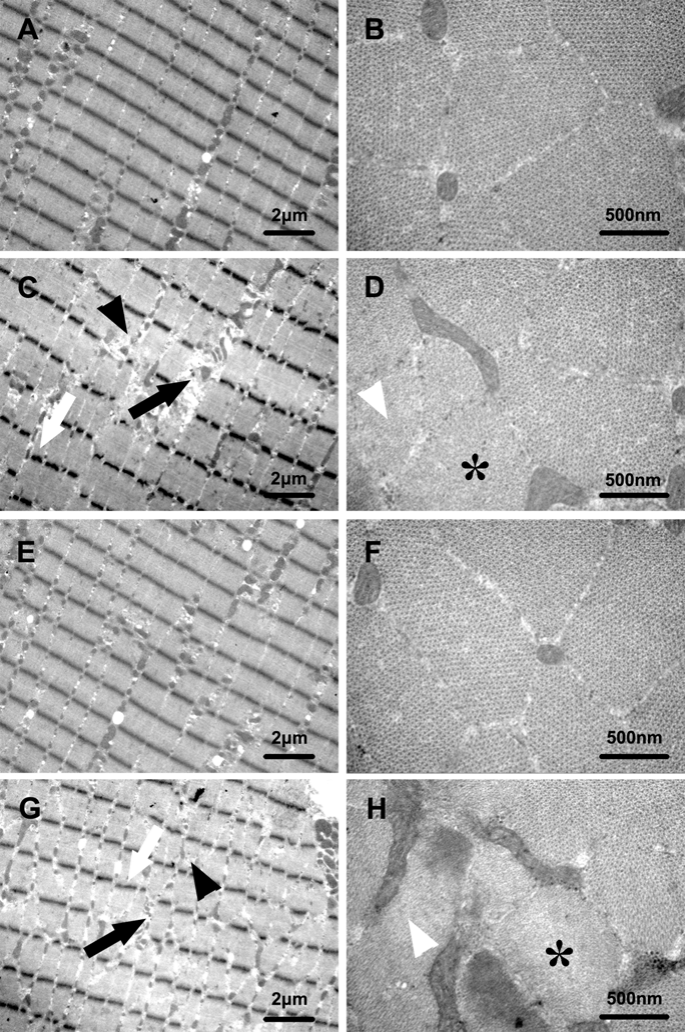
**Effects of HU in different seasons on SOL ultrastructure in Daurian ground squirrels.** Ultrastructure of longitudinal (A,C,E,G) and transverse (B,D,F,H) sections of soleus muscle in Autumn-control (A,B), Autumn-HU (C,D),Winter-control (E,F) and Winter-HU (G,H), showing several anomalies in the SOL ultrastructure of the two HU groups: disruptions of the sarcomeres with widening of the inter-fiber spaces (black arrows); irregular and discontinuous Z line (white arrows); hardly recognizable A and I bands and Z (black arrowheads); relative excess of actin filaments and decreases in amount of myosin filaments (white arrowheads); disruptions in the hexagonal arrangement (asterisks).

Compared with the corresponding control groups, the SOL ultrastructure of the two HU groups exhibited several anomalies (Table S1): (i) severe disruptions of the sarcomeres with widening of the inter-fiber spaces; (ii) Z line becoming irregular and often discontinuous; (iii) A and I bands and Z line hardly recognizable in some fibers (Fig. 4A,C,E,G); (iv) accumulation of collagen fibrils and relative excess of actin filaments; and (v) decreases in amount of myosin filaments, accompanied with disruptions in the hexagonal arrangement (Fig. 4B,D,F,H).

**Fig. 5.**
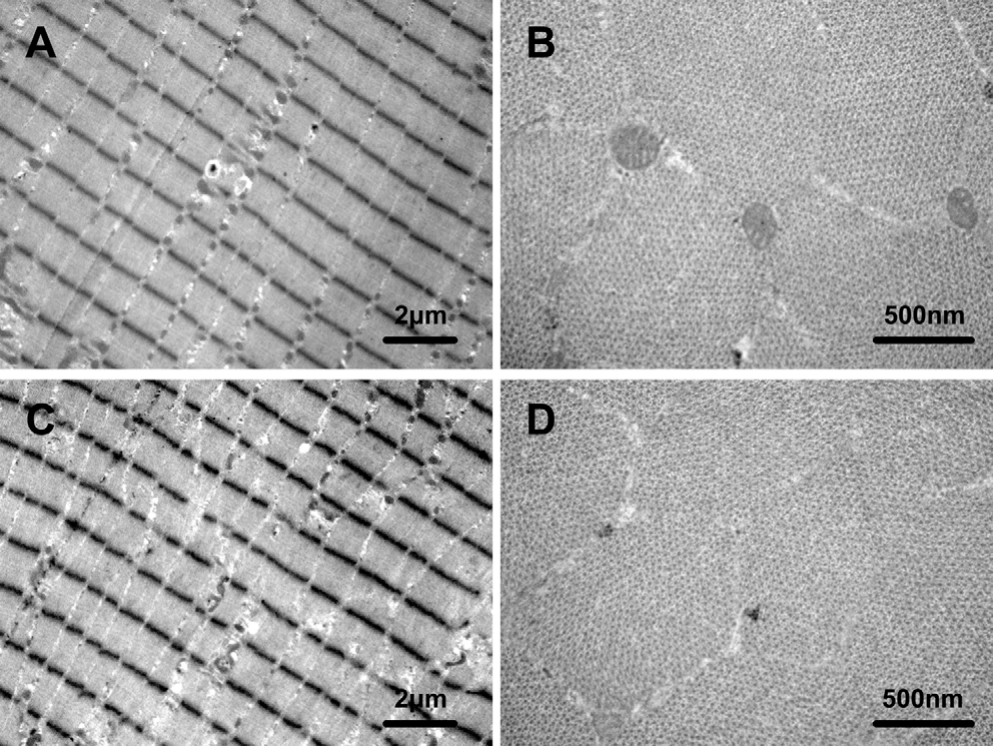
**Effects of hibernation on SOL ultrastructure in Daurian ground squirrels.** Ultrastructure of longitudinal (A,C) and transverse (B,D) sections of soleus muscle in Pre-hibernation (A,B) and Hibernation (C,D) groups, showing well preserved ultrastructure in the Hibernation group with no obvious difference from the Pre-hibernation group.

Compared with the Pre-hibernation group, the ultrastructure of the Hibernation group did not differ and was actually well preserved (Table S1): neighboring myofibers showed intact contractile structure, A and I bands, and well recognizable Z line; Z line continued throughout the entire width of myofibers (Fig. 5A,C) with easily recognizable tetragonal organization; and, myosin filaments maintained a clear hexagonal arrangement (Fig. 5B,D).

It has been well documented that in the case of muscle atrophy, abnormal ultrastructure changes occur (Hikida et al., 1989; Leivo et al., 1998; Riley et al., 2000). Therefore, our results further confirmed that SOL was atrophied after 14-day HU, but well protected during hibernation.

### Effects of hindlimb unloading in different seasons and hibernation on Akt/mTOR/FoxO1 signaling pathways in SOL muscle of Daurian ground squirrels

**Fig. 6.**
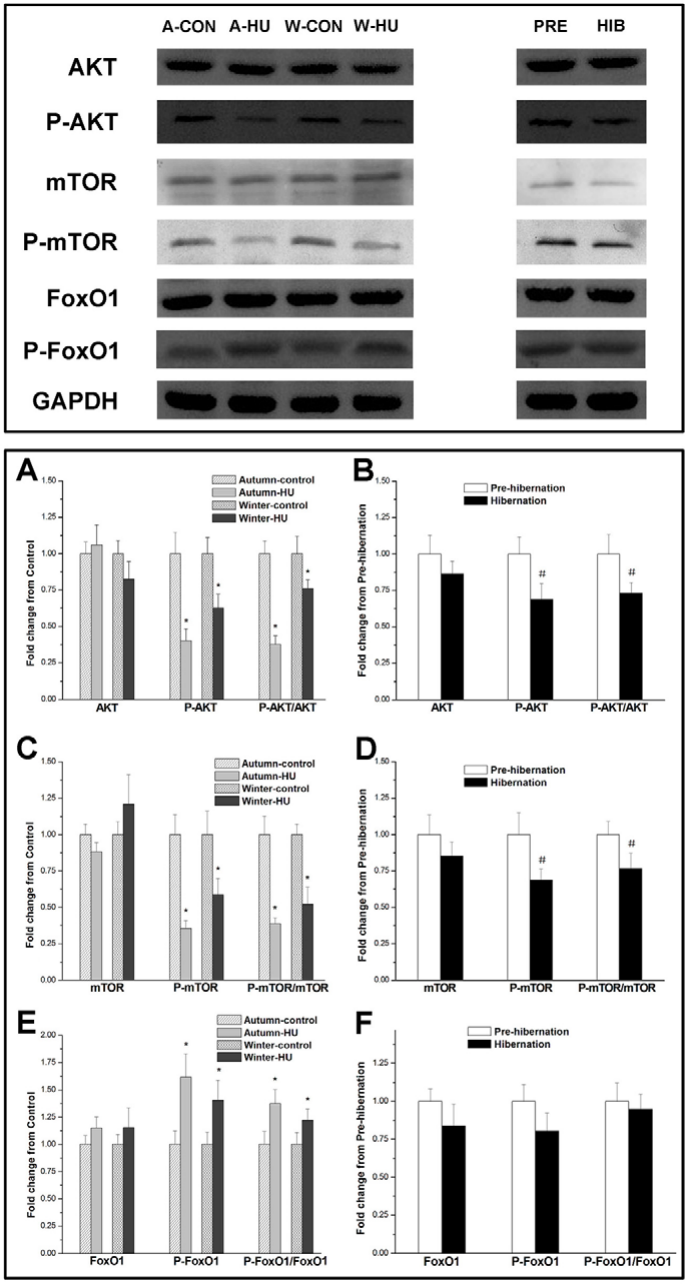
**Effects of HU in different seasons and hibernation on Akt/mTOR/ FoxO1 signaling pathways in SOL muscle of Daurian ground squirrels.** Upper panel: western blot of protein expression of Akt/mTOR/FoxO1 signaling pathway proteins after HU in different seasons and during hibernation. (A-F) Quantification of fold-changes in levels of protein expression and phosphorylation of AKT (A,B), mTOR (C,D) and FoxO1 (E,F) after HU in different seasons and during hibernation. HU significantly decreased the phosphorylation levels of Akt and mTOR but increased the phosphorylation level of FoxO1, while the phosphorylation levels of Akt and mTOR were decreased during hibernation. All protein expression data were normalized to GAPDH. A-CON, Autumn-control; A-HU, Autumn-HU; W-CON, Winter-control; W-HU, Winter-HU; PRE, Pre-hibernation; HIB, Hibernation. One-way ANOVA was used for comparisons between HU and control groups and between Autumn-control and Winter-control groups. Pre-hibernation versus Hibernation were analyzed using two-tailed independent sample *t*-tests. *P<0.05, compared with control group; #*P*<0.05, compared with Pre-hibernation. Data are expressed as means±s.d.

We investigated effects of HU and hibernation on Akt/mTOR/FoxO1 signaling pathways. The protein expression levels of total Akt, mTOR and FoxO1 remained unchanged after 14 days of HU in autumn and winter and during hibernation. HU decreased the phosphorylation levels of Akt and mTOR (Autumn-HU: −59% and −64%, *P*<0.05, respectively; Winter-HU: −37% and −41%, *P*<0.05, respectively) and increased the phosphorylation level of FoxO1 (Autumn-HU: 62%, *P*<0.05; Winter-HU: 40%, *P*<0.05). There were no statistical differences in protein expression and phosphorylation levels of Akt, mTOR and FoxO1 between the Autumn-control and Winter-control groups (Fig. 6A,C,E). During hibernation, the phosphorylation levels of Akt and mTOR were decreased as well (−31%, and −32%, respectively, *P*<0.05). The phosphorylation level of the FoxO1, however, remained relatively stable (Fig. 6B,D,F).

### Effects of hindlimb unloading in different seasons and hibernation on atrogin-1 and MuRF1 mRNA expression in SOL of Daurian ground squirrels

**Fig. 7.**
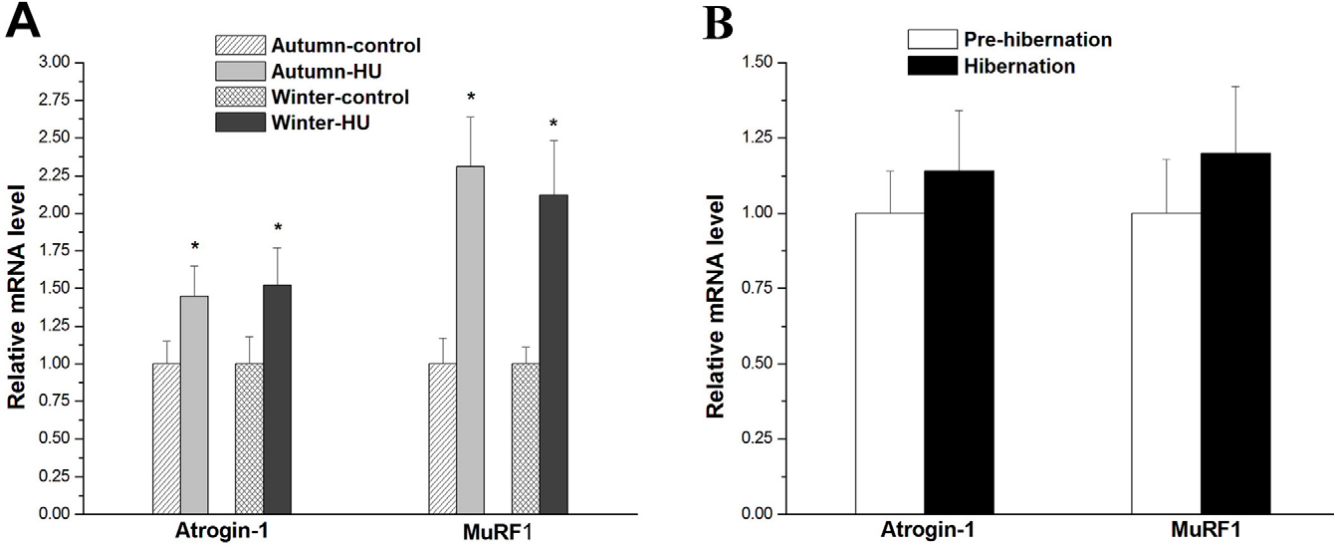
**Effects of HU in different seasons and hibernation on** ***atrogin-1*** **and** ***MuRF1*** **mRNA expression in the SOL of Daurian ground squirrels.** Changes in mRNA expression of *atrogin-1* and *MuRF1* (A) after HU in different seasons (*N*=8 each; one-way ANOVA) and (B) during hibernation (*N*=8 each; two-tailed independent sample *t*-test), showing that *atrogin-1* and *MuRF1* mRNA expression levels were upregulated after HU, but remained stable during hibernation. mRNA expression of *atrogin-1* and *MuRF1* were normalized to *GAPDH* expression. *P<0.05, Compared with control group. Data are expressed as means±s.d.

We also investigated the changes in mRNA levels of muscle-specific E3 ligases atrogin-1 and MuRF1. The mRNA expression levels of atrophy genes *atrogin-1* and *MuRF1* were normalized to housekeeping gene *GAPDH*. HU induced significant increases in *atrogin-1* and *MuRF1* mRNA expression (Autumn-HU: 45% and 52%, respectively, *P*<0.05; Winter-HU: 112% and 131%, respectively, *P*<0.05) (Fig. 7A). By contrast, there were no significant differences in *atrogin-1* and *MuRF1* mRNA expression between the Autumn-control and Winter-control groups, or between the Pre-hibernation and Hibernation groups (Fig. 7B).

## DISCUSSION

To investigate the putative factors involved in triggering the protective mechanism and the signaling pathways implicated, we performed a comparison between experimental HU and unrestrained hibernation. Recent studies have demonstrated that despite differences in the pace of atrophy across hibernation in ground squirrels (*Spermophilus lateralisand* and *Ictidomys tridecemlineatus*), muscle atrophies, if any, occur within the first two months of hibernation (Nowell et al., 2011; Hindle et al., 2015). Therefore, conducting the experiment during two months of hibernation maximized the chances of observing altered muscle biochemistry related to atrophy and investigating the underlying mechanism preventing muscle atrophy.

Our results showed that autumn and winter HU lowered SOL muscle wet mass and muscle-to-body mass ratio, decreased type I and II CSA and induced several changes in muscle ultrastructure. These findings suggest that the SOL muscles of Daurian ground squirrels were significantly atrophied during autumn and winter HU. However, SOL muscle wet mass, muscle-to-body mass ratio, type I and II CSA, fiber distribution and muscle ultrastructure remained unchanged after two months of hibernation compared with pre-hibernation, implying that a protective mechanism existed in the slow-twitch SOL, which prevented muscle atrophy. This finding is consistent with previous studies (Nowell et al., 2011; Hindle et al., 2015). Interestingly, compared with the rats in previous reports (Picquet and Falempin, 2003; Guillot et al., 2008), the type I percentage of the SOL muscle of Daurian ground squirrels was lower. The lower type I percentage suggests higher glycolytic capacity of the SOL, which may influence its sensitivity to disuse.

Limited or no loss of skeletal muscle mass occurs in hibernators even though they experience prolonged periods of inactivity during hibernation (Gao et al., 2012; Bodine, 2013b). Given this, we initially hypothesized that preventing muscle atrophy might be a natural response of hibernators to disuse, i.e. disuse is a potential factor to trigger the protective mechanism. However, the SOL atrophy induced by 14-day HU observed in our study failed to support this notion.

It has been reported that the changes in muscle mass and myosin isoform expression of ground squirrel muscles (*Spermophilus lateralis*) are seasonally induced (winter) (Nowell et al., 2011). Therefore, an alternative hypothesis might be that the protective mechanism is seasonally activated in winter. In the present study, however, Winter-HU, like Autumn-HU, induced SOL atrophy. This suggests that seasonality may not be a sufficient factor to initiate this mechanism. Therefore, other physiological alterations occurring during hibernation should also be considered as potential contributors for the initiation of the protective mechanism, especially low body temperature. Research on two species of prairie dog that use different hibernation strategies revealed that their ability to maintain muscle mass does not appear to be related to low core temperature (Cotton and Harlow, 2010). For ground squirrels, Nowell et al. (2011) found that there were no significant differences between the mass of muscles from shallow torpor at 20°C versus those in deep torpor at 4°C. Despite this, contributions of low body temperature should not be excluded completely, because a drop from euthermy at 37°C to torpor at 20°C may contribute to the maintenance of muscle mass. Further studies are needed to determine the temperature coefficient (Q10) effect on muscle mass preservation in hibernating mammals.

The Akt-mTOR pathway is the principal signaling pathway controlling protein synthesis. A major upstream regulator of the mTOR signaling pathway is Akt, the activation of which can induce mTOR phosphorylation, thus activating its downstream signals (Song et al., 2005; Zanchi and Lancha, 2008). In the present study, the activity of the Akt-mTOR pathway was suppressed both in the atrophied SOL induced by 14-day HU and in the protected SOL during hibernation, suggesting that the Akt-mTOR pathway may not participate in the protective mechanism against SOL atrophy in hibernating ground squirrels. Consistent with our observations, previous studies showed that Akt-mTOR activity was suppressed in skeletal muscles during disuse due to hindlimb-unloading (Haddad et al., 2006; Bodine, 2013a) and inactivity during hibernation (Lee et al., 2010; Wu and Storey, 2012). Considering maintaining protein synthesis is involved in preserving muscle mass during hibernation inactivity (Fedorov et al., 2014; Hindle et al., 2015), it is conceivable that other signals, except the Akt-mTOR pathway, were involved in the maintenance of protein synthesis in the SOL muscles of hibernating ground squirrels. Alternative signaling pathways should be investigated in future research.

atrogin-1 and MuRF1 are two ubiquitin ligases and atrophy-related genes involved in ubiquitination and proteolysis of muscle proteins (Glass, 2003; Sacheck et al., 2004; Bodine, 2013a). *atrogin-1* and *MuRF1* mRNA expression levels are upregulated in the skeletal muscles of non-hibernating mammals under conditions of unloading and inactivity, which facilitate the ubiquitinylation of muscle proteins, therefore increasing their degradation by proteasome (Bodine et al., 2001b; Sandri et al., 2004; Clarke et al., 2007; Cohen et al., 2009). By contrast, the maintenance of *atrogin-1* and *MuRF1* mRNA expression and the depression of proteolytic activities of proteasome have been observed in the muscles of hibernating mammals protected from atrophy (Velickovska et al., 2005; Velickovska and van Breukelen, 2007; Lee et al., 2010; Andres-Mateos et al., 2013). Consistent with these, our current study also demonstrated that *atrogin-1* and *MuRF1* mRNA expression remained unchanged in SOL through the prolonged periods of hibernation inactivity, but significantly upregulated after 14-day HU. The difference in mRNA expression of *atrogin-1* and *MuRF1* between hibernation and HU suggests they might play a role in the protective mechanism: stable atrogin-1 and MuRF1 expression facilitates muscle mass preservation via controlling ubiquitination of muscle proteins during hibernation.

It is worth noting that after 14-days, HU *atrogin-1* and *MuRF1* mRNA expression levels were upregulated, while the phosphorylation level of FoxO1 was significantly increased. Phosphorylation of the FoxO family leads to cytoplasmic retention and inhibition of its nuclear translocation activating transcription factors, in turn, block the upregulation of muscle-specific E3 ligases atrogin-1 and MuRF1 (Schiaffino and Mammucari, 2011; Bonaldo and Sandri, 2013). Therefore, it can be speculated that FoxO1 is not a key regulator elevating atrogin-1 and MuRF-1 expression in atrophied SOL muscle of ground squirrels. Another feature arising from our study is the different activation of FoxO1 and Akt. It has been reported that Akt can control the activation of FoxO transcription factors (Glass, 2003; Bonaldo and Sandri, 2013). In the present study, however, with the activations of Akt being decreased, the phosphorylation levels of FoxO1 were increased and unchanged in SOL muscles from HU and hibernating ground squirrels, respectively. Similar divergent findings were observed in thirteen-lined ground squirrels (*Spermophilus tridecemlineatus*) and bats (Cai et al., 2004; Lee et al., 2010). Growing evidence suggests that changes in the activity of FoxO transcription factors are not necessarily correlated with altered Akt activity. For example, as a component of the Skp/Cullin/F-box (SCF) ubiquitin ligase complexes, the F-box protein S-phase kinase-associated protein 2 (Skp2) is a potential candidate for the regulation of FoxO1 (Huang et al., 2005). In addition, recent studies have shown serum/glucocorticoid-induced kinase 1 (SGK1) regulates FoxO3a factor (Andres-Mateos et al., 2013).

In conclusion, the present study conducted 14-day HU in Daurian ground squirrels in autumn and winter so as to compare experimental HU and unrestrained hibernation. Our study showed that the SOL was atrophied after autumn and winter 14-day HU, but protected during two-month hibernation, which suggests that disuse and seasonality may not be sufficient to initiate the innate protective mechanism that prevents SOL atrophy during prolonged periods of hibernation inactivity. The Akt-mTOR pathway was suppressed under two conditions of disuse (HU versus hibernation), indicating its limited role in the protective mechanism. In contrast to the upregulation after HU, *atrogin-1* and *MuRF1* mRNA expression remained unchanged during hibernation, suggesting that the stable expression of atrogin-1 and MuRF1 may play a role in preventing SOL atrophy via controlling ubiquitination of muscle proteins during hibernation.

### Limitations and perspectives

In the current study, the ground squirrels experienced one to two months acclimation in cages for subsequent experiments, including hindlimb-unloading and induction of hibernation. Given that we used wild-caught ground squirrels, the effects of captivity must be considered. Cage restriction can lead to a reduction in ground squirrel locomotion, which might affect their ability to prevent disuse muscle atrophy during hibernation. In future research, this acclimation period should be shortened to less than one month to minimize captivity effects. Furthermore, since the present study investigated only slow SOL muscle, follow-up studies on other types of muscles are needed to fully understand the strategy for preventing muscle atrophy in hibernators.

## MATERIALS AND METHODS

### Animals and experimental procedure

The collection and care of the Daurian ground squirrels was conducted as previously described (Gao et al., 2012; Yang et al., 2014). Briefly, adult animals of both sexes were captured in Wei Nan, Shaanxi Province of China, in late August. Animals were transported to the Northwest University and then housed by sex (two animals) in plastic cages (0.55×0.4×0.2 m^3^) in a colony room with an ambient temperature of 18-25°C and a 12 h:12 h light-dark cycle. Animals were supplied with standard rodent chow and water *ad libitum*, supplemented with fresh fruit and vegetables. After experiencing one to two months of acclimation, 40 animals (26 females, 14 males) were carefully weight-matched and randomly assigned to five groups (*n*=8): Pre-hibernation (Autumn-control): Active animals investigated during late-autumn (also the control group of Autumn-HU); Hibernation: Animals hibernating for two months during winter; Winter-control: Non-hibernating animals in winter who were kept active in a temperature-controlled environment (20-25°C); Autumn-HU: Animals underwent hindlimb unloading in late-autumn; Winter-HU: Animals underwent hindlimb unloading in a temperature-controlled environment (20-25°C) during winter.

The HU animals received treatment as described earlier (Morey, 1979; Morey-Holton and Globus, 2002; Wang et al., 2007): the cleaned and dried tail of the animal was wrapped in adhesive plaster. The cast was linked by a hook to the top of the cage; the swivel joint of the hook permitted 360° rotation and was adjusted to allow the animals to gain food and water by moving on their forelimbs. Suspended animals were constantly kept at a 30° head-down angle. All animal care protocols were approved by the Laboratory Animal Care Committee of the China Ministry of Health.

Autumn 14-day HU was carried out in early October. According to several years of observation, the Daurian ground squirrels enter torpor in late November in our lab. Therefore, winter HU was carried out during the first half of November at 20-25°C to prevent the animals from entering shallow torpor. Daily observations were performed to record food consumption and animal state. Body temperature was monitored using a visual thermometer (Thermal Imager Ti125, Fluke, USA). During the 14-day HU period, the Winter-control and Winter-HU groups were maintained in an active state with normal feeding, and did not show typical torpor behaviors such as reduced activity, curled posture or low body temperature. The Winter-control group was sacrificed when winter HU was ended.

Induction of hibernation in ground squirrels was carried out as previously described (Gao et al., 2012; Yang et al., 2014). Briefly, the Pre-hibernation (Autumn-control) group was sacrificed in late October when autumn HU was ended. The Hibernation group was moved into a 4-6°C dark hibernaculum in mid-November. In addition to normal daily observation, we monitored the entire hibernation process using an infrared monitoring system. Body temperature was monitored using a visual thermometer to determine the time at which the animals entered torpor (Fig. S1). After experiencing two-month hibernation, the Hibernation group was sacrificed.

### Sample preparation

After animals were anesthetized with sodium pentobarbital (90 mg/kg ip), both SOL muscles were collected, and body mass and muscle wet mass were then recorded. One SOL muscle was prepared for histochemical and ultrastructural analysis, and another one was frozen in liquid nitrogen and stored at −70°C until further assays. All experimental protocols were reviewed and approved by the Northwest University Ethics Committee.

### Immunohistochemistry

Samples (5 mm) from the SOL mid-belly were embedded with an optimal cutting temperature gum (Sakura Finetek USA Inc., USA), and then immediately frozen in isopentane pre-cooled in liquid nitrogen. Serial 10 µm transverse sections were cut in a cryostat (Leica, Wetzlar, CM1850, Germany) and mounted onto poly-L-lysine coated slides. Myosin heavy chain II (MHC II) was stained by SABC immunohistochemical staining method using antibody MY32 (Boster, China). The antibody was diluted at 1:100 with phosphate-buffered saline (PBS) solution. Serial cross sections (10 µm) were incubated in methanol-hydrogen peroxide solution (0.3% hydrogen peroxide) for 30 min. After washing three times for 5 min each, sections were blocked with goat serum diluted in PBS for 30 min at 37°C. The sections were incubated with primary antibodies for 2 h at 37°C followed by rinsing with PBS solution. Subsequently, the sections were incubated with biotinylated universal secondary antibody for 60 min at 37°C and then washed with PBS three times for 5 min each. Peroxidase conjugated streptavidin-biotin complex (Boster) was used for 30 min at 37°C. After another three washing steps with PBS, diaminobenzidine (DAB) (Boster) was added to visualize the sections. At the end of staining, the slides were dehydrated with alcohol and dimethylbenzene, and then mounted with Eukitt. In the corresponding negative control assays, the primary antibody was replaced with PBS.

SOL muscle fiber distribution and CSA analyses were carried out as previously described (Gao et al., 2012). Briefly, muscle typing was established from at least 300 fibers per muscle. Fiber distribution was expressed as the number of all fibers of each type relative to the total number of fibers. The CSA of at least 100 fibers per muscle was measured using a Quantimet-570 video image analyzer (Leica, UK).

### Transmission electron microscopy (TEM)

Small pieces of SOL samples were immediately fixed with 2.5% glutaraldehyde in 0.1 M phosphate buffer for 2 h and postfixed with 1% osmium tetroxide in 0.1 M phosphate buffer for 2 h. Subsequently, the samples were dehydrated in graded ethanol and embedded in Epon 812 epoxy resin (Electron Microscopy Sciences, USA). Longitudinal and transverse semi-thin sections were obtained using a semi-thin slicer (Reichert, USA). After staining with toluidine blue, the orientation-adjusted sections were sliced by an ultramicrotome (LKB-NOVA, USA). Following double-staining with 160 Reynolds lead citrate (Reynolds, 1963) and ethanolic uranyl acetate, the ultrathin sections were examined using a transmission electron microscope (JEM-100SX, Japan).

### Western blot analysis

Total proteins were extracted from SOL muscle using RIPA lysis buffer (Beyotime Institute of Biotechnology, China) with a protease inhibitor tablet cocktail (Pierce Chemical Co., USA) accorded to the manufacturer's guidelines. The BCA assay was performed to determine the protein concentration. Total proteins (40 μg) from each SOL sample were separated by 6–15% SDS-PAGE gels (depending on the molecular weight of the protein). The gels were then transferred to polyvinylidene fluoride (PVDF) membranes (Millipore, USA) using Bio-Rad semi-dry transfer apparatus (Bio-Rad Laboratories, USA). Following transfer, membranes were blocked with 5% skim milk dissolved in TBST (10 mM Tris-HCl, 150 mM NaCl, 0.05% Tween-20, pH 7.6) for 2 h at room temperature.

The membranes were incubated with primary antibodies to total and phosphorylated proteins, including rabbit mAb anti-Akt (1:1000, Cell Signaling Technology, USA, #2920), rabbit mAb anti-p-Akt^Ser473^ (1:1000, Cell Signaling Technology, #4060), rabbit mAb anti- mTOR(1:500, Cell Signaling Technology, #2983), rabbit mAb anti-p-mTOR^Ser2448^ (1:500, Cell Signaling Technology, #2971), rabbit mAb anti-FoxO1 (1:000, Cell Signaling Technology, #2880), rabbit mAb anti-p-FoxO1^Ser256^ (1:800, Cell Signaling Technology, #9461) and rabbit polyclonal anti-GAPDH (1:500, Santa Cruz Biotechnology, USA, sc-25778). Following washing, the membranes were incubated with HRP-conjugated anti-rabbit secondary antibodies (Pierce Chemical Co.) for 1 h at 37°C. After another washing, immunoblots were visualized using enhanced chemiluminescence (ECL) reagents (Pierce Chemical Co.) according to the manufacturer's protocols. Quantity One software (Bio-Rad Laboratories) was used for quantification. All protein expression data were normalized to GAPDH. Fold-change of every target protein was calculated (relative to Control/Pre-hibernation).

### Real-time PCR

Total RNA from muscle samples was extracted using RNAiso Plus reagent (Takara Bio, China) in accordance with the manufacturer's guidelines. We used 1% agarose gel electrophoresis to examine RNA integrity. A Nano-Photometer (Implen, Germany) was used to confirm purity and concentration of RNA samples, and samples with OD260/280 of 1.8-2.0 were acceptable. Total RNA was converted into cDNA by reverse transcription using PrimeScript RT Master Mix (Takara Bio). All primers were designed by PrimerQuest (http://www.idtdna.com/Scitools/Applications/Primerquest/) to produce exon-spanning amplicons. Gradient PCR followed by 2% agarose gel electrophoresis was performed to verify the product size, specificity of the primers and most appropriate annealing temperature (Fig. S2). All primers were tested for efficiency by serial dilution of cDNAs. The efficiencies of the target and reference gene amplifications were between 90-110% and approximately equal. Real time PCR was performed with LightCycler 480 (Roche Applied Science, Germany) using SYBR Premix Ex Taq-II (Takara). The PCR mixture included 10 μl of SYBR Premix Ex Taq-II (2×), 2 μl of the diluted single-stranded cDNA, 1.6 μl of gene-specific primers set (0.8 μM), and 6.4 μl sterile dH2O in a final volume of 20 μl. The PCR thermocycling protocol was as follows: Initial denaturation at 95°C for 5 min, 40 cycles of 30 s at 95°C and 40 s at 58°C. Gene-specific PCR was performed in duplicate for each cDNA sample. Melt curve analysis displaying a single sharp peak confirmed specificity of primer annealing (Fig. S3). The mRNA expression of each target gene was normalized to *GAPDH* in each sample, and quantitation of gene expression was determined by the equation 2^−ΔΔCT^. The gene-specific primers were as follows: *atrogin-1*, forward, 5′-AGACCGGCTACTGTGGAAGAG-3′, reverse, 5′-CCGTGCATGGATGGTCAGTG-3′, *MuRF1*, forward, 5′-GCCATCCTGGACGAGAAGAA-3′, reverse, 5′-CAGCTGGCAGCCCTTGGA-3′; *GAPDH*, forward, 5′-GACAACTTTGGCATCGTGGA-3′, reverse, 5′-ATGCAGGGATGATGTTCTGG-3′.

### Statistical analysis

All data were analyzed using SPSS 10.0 (SPSS Inc., Chicago, IL, USA) and expressed as means±s.d. Significant differences between HU and control groups and between Autumn-control and Winter-control groups were examined using one-way ANOVA with Bonferroni post-hoc tests. Pre-hibernation versus Hibernation were analyzed using two-tailed independent sample *t*-tests. Statistical significance was set at *P*<0.05.
