# Correction: Stable *atrogin-1* (*Fbxo32*) and *MuRF1* (*Trim63*) gene expression is involved in the protective mechanism in soleus muscle of hibernating Daurian ground squirrels (*Spermophilus dauricus*)

**DOI:** 10.1242/bio.059737

**Published:** 2022-11-30

**Authors:** Kai Dang, Ya-Zhao Li, Ling-Chen Gong, Wei Xue, Hui-Ping Wang, Nandu Goswami, Yun-Fang Gao

There was an error published in *Biol. Open* (2016) **5**, 62-71 (doi:10.1242/bio.015776).

In Fig. 4, the Autumn-control image in panel B was erroneously a duplication of the Autumn-HU image in panel D.

**Fig. 4:**
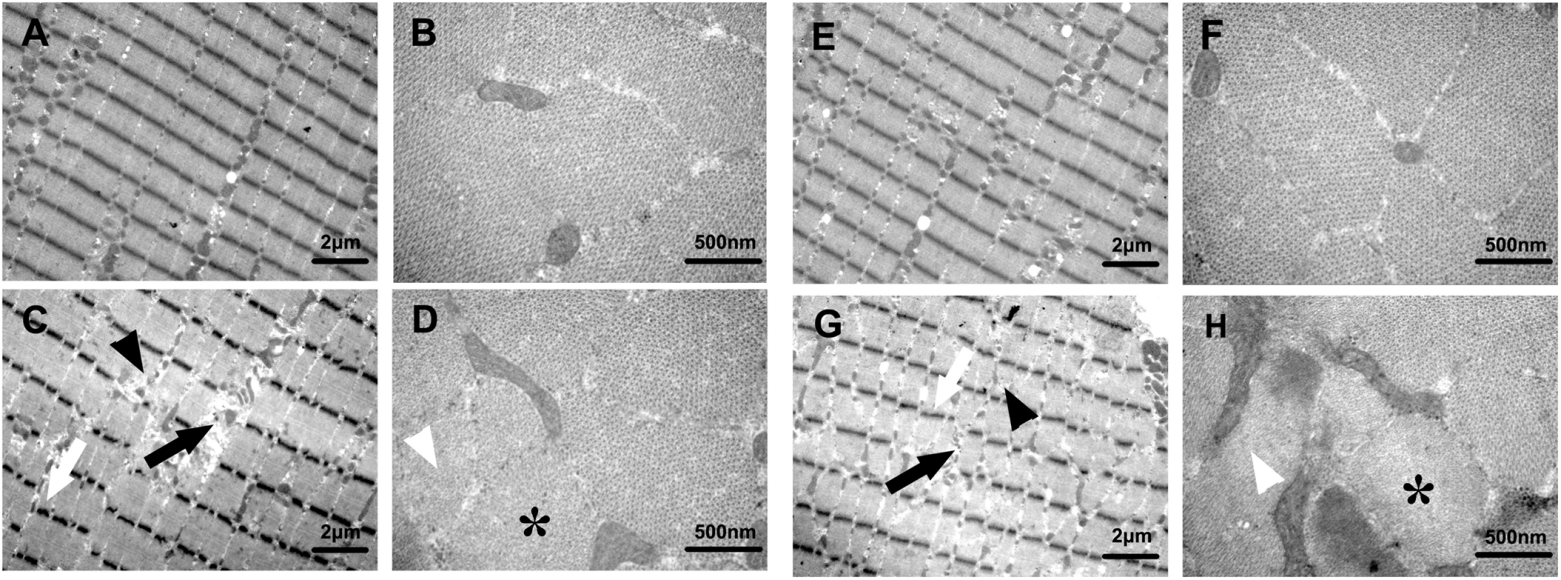
**(corrected). Effects of HU in different seasons on SOL ultrastructure in Daurian ground squirrels.** Ultrastructure of longitudinal (A,C,E,G) and transverse (B,D,F,H) sections of soleus muscle in Autumn-control (A,B), Autumn-HU (C,D),Winter-control (E,F) and Winter-HU (G,H), showing several anomalies in the SOL ultrastructure of the two HU groups: disruptions of the sarcomeres with widening of the inter-fiber spaces (black arrows); irregular and discontinuous Z line (white arrows); hardly recognizable A and I bands and Z (black arrowheads); relative excess of actin filaments and decreases in amount of myosin filaments (white arrowheads); disruptions in the hexagonal arrangement (asterisks).

The corrected figure is shown below.

The authors apologise to readers for this error, which does not impact the results or conclusions of this paper.

